# Agro-Food Byproducts as a New Source of Natural Food Additives

**DOI:** 10.3390/molecules24061056

**Published:** 2019-03-18

**Authors:** Margarida Faustino, Mariana Veiga, Pedro Sousa, Eduardo M. Costa, Sara Silva, Manuela Pintado

**Affiliations:** CBQF–Centro de Biotecnologia e Química Fina–Laboratório Associado, Escola Superior de Biotecnologia, Universidade Católica Portuguesa, Rua Arquiteto Lobão Vital 172, 4200-374 Porto, Portugal; afaustino@porto.ucp.pt (M.F.); mveiga@porto.ucp.pt (M.V.); pedro.miguel.c.s@hotmail.com (P.S.); emcosta@porto.ucp.pt (E.M.C.); snsilva@porto.cup.pt (S.S.)

**Keywords:** byproducts, food additives, antimicrobial, antioxidant, colorants, texturizing agents, foaming capacity and emulsifiers

## Abstract

Nowadays, the agro-food industry generates high amounts of byproducts that may possess added value compounds with high functionality and/or bioactivity. Additionally, consumers’ demand for healthier foodstuffs has increased over the last years, and thus the food industry has strived to answer this challenge. Byproducts are generally secondary products derived from primary agro-food production processes and represent an interesting and cheaper source of potentially functional ingredients, such as peptides, carotenoids, and phenolic compounds, thus promoting a circular economy concept. The existing body of work has shown that byproducts and their extracts may be successfully incorporated into foodstuffs, for instance, phenolic compounds from eggplant can be potentially used as a mulfitunctional food additive with antimicrobial, antioxidant, and food colorant properties. As such, the aim of this review is to provide insights into byproducts and their potential as new sources of foodstuffs additives.

## 1. Introduction

Food functionalization is an ever-increasing market that requires new bioactive ingredients that can be used by the food industry for the development of innovative functional products with scientifically sustained claims. In this regard, much attention has been paid in recent years to natural compounds and their associated bioactivities. However, natural sources are finite, and new alternatives have to be sought to sustain the ever-growing needs for ingredients and additives of the food industry [1,2]. 

The European Union (EU) action plan for the circular economy to reduce food waste includes a strategic approach based on the reduction, reuse, recovery, and recycling of materials and energy, enhancing the value and consequently the useful life of products, materials, and resources in the economy. The reuse of agro-industrial byproducts can represent a renewable source for some already in use food additives or even originate new added-value ingredients with functional compounds and properties, which will benefit the entire food system [3]. For instance, byproducts contain polysaccharides, organic acids, proteins, and other compounds, which, at no additional production cost and at a reduced industrial cost, make them a rich source of natural compounds that can potentially be applied in the food industry as food additives sources (summarized in Table 1) [4]. 

Furthermore, these natural compounds may also be regarded as nutraceutical ingredients or complements, allowing for the development of products with enhanced nutritional value, potential health benefits, longer shelf-life, as well as a good sensory profile [5,6,7]. 

Taking this into account, this review aims to provide a broader look into the potential use of byproducts as new sources of food additives (already in use or potential new ones) to be used by the industry.

## 2. Consumer Perspectives

In the 1960s, the E number system was introduced to assure consumers that the additives included into their foodstuffs were safe for consumption. However, the use of this code made some consumers reticent in regard to these compounds with false allegations (on their lack of safety) being made in some publications [37,38,39]. Moreover, with the increase in life expectancy, concerns grew in regard to overall life quality. This, coupled with the widespread link between diet and health, made consumers particularly aware of the foodstuffs they ingested and increased the demand for healthier solutions. One trend associated with this shift in perception is “clean labels”, i.e., products that are perceived as “natural”, such as “free-range”, “less processed”, “organic”, or “biological” foods [39,40]. Overall, this means that not adding additives has become a differentiating factor for food products, and consequently that the industry has become more interested in new solutions that, while exerting the same technological effect as traditional additives, have no negative perception. Agro-food byproducts present an interesting source of bioactive and technologically relevant compounds that, given their low commercial value, pose as a relevant potential source of new natural additives [41,42,43,44].

## 3. Applicable Legislation

Food additives have an essential role in the current industry and consumption habits, as they not only make food products more appealing, but they increase their stability and inherent safety. Overall, food additives may be defined as compounds/extracts that are added to a food product in order to accomplish a specific technological goal but are not ingested as a food product themselves. According to the European Food Safety Agency (EFSA), an additive must not pose a safety concern for the consumers health (when ingested) while fulfilling a specific technological need that cannot be satisfied through other reasonable means. Examples of these needs are the enhancement of the sensory quality, the fulfillment of specific dietary needs, or the ease of production, packaging, transport, and/or storage of food products [45]. Overall in the EU, the use of additives (non-enzymatic) is regulated by European Commission (EC) No 1333/2008 with the additives, the list of allowed additives, and subsequent limitations always dependent on the appearance of new evidence regarding their safety. In this legislation, the different groups are defined (Table 2) along with rules on how an additive must be referred to in a product (e.g., the information must be present in the label with the compounds referred to either by their name or their E-number and by the function they play in the final product). Moreover, food additives must follow specific purity criteria that are described in three different directives: Directive 2008/60/EC for sweeteners; Directive 2008/128/EC for colors; and Directive 2008/84/EC for other additives [46,47,48]. After the inclusion of the list of approved additives and food carriers (and the conditions associated with their use) into Regulation (EC) No 1333/2008, a revision of the purity criteria of food additives was undertaken, resulting in a new regulation, Regulation (EU) No 231/2012, that repealed the previous directives for sweeteners, colors, and other additives [45,48,49,50].

Some of the additives currently allowed under the scope of Regulation (EC) No 133/2008 may be found in agro-food byproducts. Namely, anthocyanins (E163) may be found in grape/winemaking byproducts, chlorophylls (E140) may be found in almost all green leaf vegetable byproducts or mango peels along with all green leaf wastes that result from pruning during agricultural production, and lycopene (E160d) can be found in tomato wastes [51,52,53,54,55,56,57]. There is a consensus that as long as the additive compound/molecule is already part of the list of authorized compounds, it can be used [58]. However, if the production process is varied significantly (by using a significantly different raw matter or using new production procedures), the “new” additive must be evaluated again by EFSA. This means that the focus given to the development of new and more efficient green technologies to attain additives from agro-food byproducts may result in potential new additives that must be subjected to a new evaluation in order to ensure their safety. This path starts with a thorough safety (short and long term) evaluation of any potential metabolic, genotoxic, reproductive, and chronic or carcinogenic side effects [59]. Following this, it is possible to define a no observable effect level (NOEL) and then an allowable/acceptable daily intake (ADI). Once all the relevant information is gathered, EFSA or other similar organizations (like the Food and Drug Administration—FDA) can be petitioned to validate the introduction of the additive through an amendment of the legislation in order to add the substance to the list of authorized food additives. If this authorization is granted, the additive will be eligible to be used on the market under direct supervision of the agency that granted the permission [49,59,60]. In the EU, the submission of a potential new additive for validation must start with an application submitted to the EC, who will verify it. If valid, EFSA must then give an opinion within a timeframe of nine months, a period that may be extended if further information is required from whoever submitted the application (for risk management purposes, EC may also require further elucidation). If EFSA gives a positive opinion, EC has nine months to submit a regulatory draft aimed at the inclusion of the substance in the allowed additives list, whose approval is dependent on the votes of member states. If approved, as with all decisions of the EU, it must then pass a three month long period of scrutiny. Overall, this process is very long and, in the new era of circular economy where food byproducts valorization is of the upmost importance, legislation approaches should be reanalyzed to facilitate and speed up the process of new additives approval while still guaranteeing the safety of the final additive [49,61].

## 4. Preservatives

Microbiological processes can adversely affect the quality of food, leading to its spoilage. For this to occur, conditions that favor the growth and development of spoilage microorganisms must be met, such as bioavailable nutrients, favorable water activity, adequate pH value, presence/absence of oxygen, and redox potential [62]. The term “food spoilage” is only applied if the changes in the foodstuffs due to the microorganisms’ potentially harmful metabolic products become recognizable, thus making the product unsafe for consumption and augmenting the risk of foodborne illness [62,63,64,65]. However, not all microbiological change in food is considered harmful (for example, fermentation of grape juice in order to produce wine) [66].

Taking this into account, preservatives are widely used in the food industry in order to prevent microbial contaminations, demonstrating a significant impact upon a product’s shelf-life as well as food safety [58,66,67,68]. There are different antimicrobial compounds that can potentially be used as preservatives ranging from enzymes, bacteriocins, fungicides, and salts to essential oils and other components, some of which may be found agro-food byproducts [7,69,70,71,72,73,74,75,76]. The use of natural compounds to replace traditional additives is an emerging trend that has been driven by the consumer’s preferences for “clean labels”, with the scientific community striving to provide natural alternatives, some of which may be attained from agro-food byproducts (e.g., phenolic compounds) [7,44,77]. Nitrates (E240-E259) and nitrites (E249-E250) are the most commonly used preservatives in foodstuffs. Both have been associated with the formation of nitrosamine (a carcinogenic compound responsible for the development of gastric and other types of cancer). Therefore, actions have been taken to reduce their intake [the current daily intake for nitrates is 3.7 milligrams per kilogram of body weight (mg/kg bw/day), while for nitrites it was re-established to 0.07 mg/kg bw/day] [78,79,80]. However, EFSA determined that there was insufficient evidence to ban the use of nitrates and nitrites as food additives due to health concerns, particularly with them being the only additives capable of exerting antimicrobial activity against *Clostridium botulinum* and preventing botulinic toxin production/accumulation [81]. 

Agro-food byproducts, particularly fruit peels and seeds, have been regarded as a potential source of preservatives with several reports reporting on the potential antimicrobial activity of different fruit and vegetable byproduct extracts, which could potentially be translated into an industrial application if the appropriate regulatory body gives a positive opinion [7,41,67,82]. For instance, Gul and Bakht [83] reported how an ethanolic turmeric extract possessed antibacterial activity against *Escherichia coli* and *Staphylococcus aureus,* an effect that has been attributed to its phenolic content [84,85,86]. Additionally, turmeric oil, a byproduct from curcumin manufacture, has also been described as possessing antibacterial and antifungal activity [86,87,88]. Berries are fruits with high phenolic content, particularly anthocyanins. While by themselves they possess an interesting commercial value, if the fruits fall from the bushes (overly ripe berries), they will not be commercialized [89,90,91]. However, they remain a phenolic rich fruit that can be used as a source of potential antimicrobial additives. For instance, blueberry and cranberry anthocyanin-rich extracts have been reported as possessing vast antimicrobial activity and could potentially be exploited as new natural food additives [92,93,94,95,96,97]. Olive leaves are also a good source of phenolic compounds and have been reported as possessing some antimicrobial activity against *Bacillus cereus*, *E. coli*, *S. aureus,* and some fungi such as *Candida albicans* and *Cryptococcus neoformans* [98,99,100,101]. Wang, et al. [102] reported how the addition of green tea polyphenols (mainly constituted by catechins) and tocopherol to dry-cured bacon resulted in significantly lower *Enterobacteriaceae* content. Green tea and black tea wastes have been studied for their potential nutritive, antimicrobial, and antioxidant values due to their high tannin and catechin content [103,104].

Wine pomace, a well-known byproduct, also showcases some potential as a new source of antimicrobial food additives, as its activity has also been associated with its high phenolic content and anthocyanins in particular [22,105]. Pomegranate peel extracts, reported to be natural inhibitors of food-borne pathogens such as *Listeria monocytogenes*, *E. coli,* and *Yersinia enterocolitica*, have been added to poultry products with the results showing good antimicrobial activity against *S. aureus* and *B. cereus* and permitting the increase of shelf-life by two weeks [32,33,106,107,108,109]. Avocado, a tropical fruit, has also been described as possessing a relevant antimicrobial activity, with several reports focusing on the biological activity of its peel and seed [110,111]. For instance, Calderón-Oliver et al. [112] reported how a nisin (an antimicrobial peptide) avocado peel mixture resulted in an enhancement of nisin’s antimicrobial activity against food-borne pathogens such as *Listeria* sp. Overall, the reported results favor the use of natural byproduct extracts as potential new preservatives at an industrial level, helping to reduce costs and environmental impact, although the leap to an industrial setting is limited by a lack of regulatory framework for their use.

Currently, the only animal derived antimicrobial additive used in the EU and United States (US) is lysozyme (E1105). Lysozyme originates from eggs, and while it is mainly used in cheese conservation, studies concerning eggs, milk, and beef have been carried out. However, it does not exert any action against yeasts or fungi [113,114,115].

## 5. Antioxidant Additives

Oxidation is a not a process exclusive to the human body. It occurs in every living organism and biological system, such as food products. Food oxidation may result in altered flavor, color, nutritional value, and texture, as well as create toxic compounds [82,116,117]. As such, antioxidant compounds are one of the most important conservation technologies used by the food industry with their main function being the prevention of oxidative induced degradation of foods, therefore allowing for extended shelf times [82,117,118]. These additives help stabilize lipids (avoiding lipidic peroxidation) as well as other compounds and can neutralize free radicals, avoiding a cascade of oxidative reactions. [117,119].

As previously mentioned, due to a shift in consumer preferences, in recent years there has been an increase in the demand for more natural (i.e., with less additives) food products [120]. As such, there have been studies comparing synthetic and natural antioxidants with results showing that natural phenolic antioxidants are capable of inhibiting oxidation and toxin formation, meaning that they present an interesting natural alternative to the traditionally used antioxidant additives [117,121]. Butylated hydroxy anisole (BHA), butylated hydroxytoluene, ethoxyquin, tert-butylhydroquinone, and propyl gallate are the most common synthetic antioxidants used in foods. Reports on their potential health impact are divided [121,122,123,124].

Since plants are one of the main sources of antioxidants compounds, agricultural byproducts are among the most relevant potential sources of natural antioxidants that could be exploited for product quality preservation. Phenolic compounds, besides being associated with antimicrobial activity, are known for their high antioxidant capacity. They are ubiquitous to plants and therefore present one interesting class of antioxidant compounds to be exploited, although other compounds with a strong antioxidant capacity can also be found, such as some vitamins (vitamin C, E, and A), bioactive peptides, polysaccharides, some minerals, and enzymes. Any byproduct with a high content of any of these compounds may be regarded as a possible source of new antioxidant food additives, e.g., overly ripe berries, or citric and exotic fruits, peels, and seeds [77,116,121,125]. Meat byproducts (including blood, bones, meat trimmings, and viscera) can result in protein hydrolysates with a relevant bioactivity, namely antioxidant bioactive peptides [126,127]. Onion byproducts (namely onion peels and stems) have been regarded as potential food additives due to their antioxidant and anti-browning properties [128]. Larrosa et al. [129] reported that adding an artichoke byproduct extract (namely artichoke blanching waters) to a tomato juice resulted in higher antioxidant activity (measured by the DPPH^•^ and ABTS^•+^ methods) and consequently a longer shelf life for this product. Similarly, eggplant aqueous acetone extracts have also been studied, with reports describing a high antioxidant potential of its peels (evaluated by FRAP and TEAC) likely due to its rich anthocyanin content [130]. Mango byproducts are an example of a vastly studied tropical fruit with a high antioxidant capacity and a wide range of potential applications [54,131]. An example is the inclusion of mango peel powder in macaroni and bakery products (such as biscuits) to provide some added functional value as well as function as a natural antioxidant (as the supplemented products exhibit a higher capacity to quench DPPH^•^) [54,132,133].

The potential for the use of natural alternatives to antioxidant additives has been supported by the work of Caleja, Barros, Antonio, Oliveira, and Ferreira [121], who reported no significant differences between the use of natural extracts (chamomile and fennel) and a synthetic (BHA) antioxidant additive in biscuits, with no significant changes in color or nutritional value observed after 60 days of storage. Similarly, there have been reports on the successful addition of natural antioxidant extracts to bakery, dairy, and meat products, which also confer some added functionality to the foodstuffs [79,121,134,135,136,137]. Overall, byproducts of industrial fruit processing consist mainly of seeds, peels, and unused flesh. Some of these residues have been reported as possessing a higher concentration of bioactive compounds than the used fruit flesh [108,111,132,138,139,140]. Furthermore, the antioxidant compounds of natural origin, when attained using adequate solvents, are considered as generally recognized as safe (GRAS). Moreover, some of the antioxidant compounds naturally found in these byproducts are already approved for use as antioxidant additives and possess an E number, namely ascorbic acid (E300), tocopherol (E306), and β-carotene (E160a) [68,102,113].

## 6. Food Colorants

Although the flavor and nutritional value tend to be the most studied and appreciated components of a food product, its appearance is also an important sensory aspect [141,142]. Colorants are food additives used to impart color to foodstuffs to make them look more appetizing and/or help compensate color loss due to exposure to natural elements (light, air, temperature, etc.) [143,144]. Color plays an important role in the consumer’s emotional reaction and acceptance of food. Color is appreciated both for its aesthetic and quality indicator role, as an adequate color is frequently used for quality assessment due to its association with flavor, nutritional value, and food safety [145]. Color provides visual suggestions to flavor identification and taste thresholds, influencing food preference, food acceptability, and ultimately, food choice [146]. Current market trends include the substitution of synthetic colorants for natural compounds found in certain foodstuffs (such as fruits) or in food byproducts, a trend that is reinforced by studies regarding possible detrimental effects of synthetic colorant usage in foods [142,144]. Most commercial colorants are produced synthetically, including erythrosine (red), cantaxanthin (orange), amaranth (azoic red), tartrazine (azoic yellow), and annatto bixine (yellow orange) [67]. Nonetheless, a few colorants like carotenoids (β-carotene, astaxanthin, canthaxanthin, and zeaxanthin) are obtained from natural sources, such as tomato, paprika, and algae [147]. However, synthetic colorants are still used due to their stability and low cost [44]. As agro-food byproducts are usually discarded, their use as a new source of these coloring agents could be a means to shift to more natural colors while still maintaining a low production cost (Table 3).

As previously mentioned, consumers have been demanding the replacement of synthetic colorants by natural alternatives. Authors like Siegrist and Sütterlin [155] reported that symbolic information such as the E-numbers on the foodstuff’s label influences a consumer’s perception of different foodstuff and its origin, with consumers being hesitant to accept the addition of synthetic food additives. Additionally, there have been several reports pertaining to synthetic colorants side effects, including hypersensitive and allergic reactions as well as potential toxicity and carcinogenicity claims [144,156,157]. Natural additives have been associated with health promoting benefits, as they are a part of the bioactive compounds present in fruit and vegetable byproducts. However, the use of these natural pigments can be limited by their lower stability and weaker color strength (when compared to their synthetic counterpart). Additionally, natural additives may confer an undesirable flavor or odor to the food products, which will negatively impact the consumer’s acceptance [142,145,158]. Nonetheless, fruit and vegetable byproducts have become an important potential source of natural pigments, as they are colored by green chlorophylls, yellow-orange-red carotenoids, red-blue-purple anthocyanins, and red betanins [158]. Overall, the main groups of coloring substances found in nature are carotenoids, anthocyanins, porphyrins, and chlorophylls [145,158,159,160].

Anthocyanins are a good example of natural color additives. These compounds are a group of natural pigments responsible for the blue, red, purple, violet, and magenta coloration of several species in the plant kingdom. They can also be found in extracts of their byproducts. Some examples are winery byproducts, radishes, red potatoes, red cabbage, black carrots, purple sweet potatoes, coffee husks, and berries, among others [106,161].

Carotenoids stand as the major group of compounds used as color additives. These natural pigments are responsible for many of the colors seen in edible fruits, vegetables, mushrooms, flowers, and even lobster and trout hues from the animal kingdom [158]. Much like anthocyanins, carotenoids are produced synthetically (β-carotene, astaxanthin, canthaxanthin, and zeaxanthin), although some are obtained from natural sources, namely annatto, marigold, tomato, algae, and microbial fermentation [157]. In addition, these compounds function as sources of provitamin A and are capable of absorbing solar light, oxygen transporters, powerful quenchers of singlet oxygen, as well as other functions not yet studied [160]. 

The natural pigments were defined in the Regulation (EC) No 1333/2008 of the European Parliament and of the Council of 16 December 2008 and are listed in the Annexes of said regulation [161]. This document includes detailed information on the application of individual pigments in defined food products, their doses, and limitations of use. Presently, 16 natural pigments are permitted: betalains–betanin, quinones–cochineal, flavonoids–anthocyanins, isoprenoids–carotene, annatto (bixin, norbixin), paprika extract, lutein, canthaxanthin, porphyrins–chlorophylls and chlorophyllins, and copper complexes of these compounds, among others, like caramels, curcumin, or plant coal. According to the Regulation (EC) No 1129/2011 [162] of the European Parliament, in the EU, there are 40 color additives legislated for food use.

New technologies such as pulsed-light, high pressure, pulsed-electric, magnetic fields high pressure processing, ionizing radiation, and ultraviolet radiation are being studied and could allow for the use of byproducts as natural source pigments, which could then be exploited as potential new food colorants in the food industry with the advantage of imparting potential health benefits to the consumer as well as contributing to an economical valorization of residues and avoiding waste [163]. For instance, there have been studies regarding the addition of banana peels to biscuits, which resulted in a product with low calories and high dietary fiber content without any significant differences in color, aroma, and taste observed. The banana peel was incorporated at a 10% and 20% concentration into the biscuits [164,165,166]. The peel is the main byproduct of the banana, rich in phytochemical compounds with high antioxidant capacity, such as phenolic compounds, anthocyanins (delphinidin and cyanidin), carotenoids (β-carotenoids, α-carotenoid, and xanthophylls), catecholamines, sterols, and triterpenes, which, as previously mentioned, could provide different functions as potential food additives besides coloring, namely as antioxidant and antimicrobial components [18,167]. Mango is another example of a fruit with biologically interesting compounds (including phenolic compounds, carotenoids, and dietary fiber) that could be used in the food industry. Most mango byproducts result from the epicarp and endocarp, but it is the mango seed kernel residue with the highest amount of carotenoids in its composition, which is likely due to the amount of fruit pulp left around the kernel by the chopping machine. The carotenoid content was found to be four to eight times higher in ripe mango peels compared to raw fruit peels [133,165]. The high levels of bioactive compounds in the mango peel makes this byproduct a potentially valuable raw material for the formulation of additives and supplements for the food industry [168]. Likewise, tomato peel is the main byproduct resulting from the tomato processing industry [169]. The carotenoid pigment lycopene present is the compound responsible for its red color. Tomato lycopene extract and tomato lycopene concentrate from tomato peels have been approved for use as colorants exempt from certification [170]. Oleoresins, powders, and water-dispersible preparations that can impart colors from yellow to orange to red are commercially available. An example of the utilization of lycopene from tomato byproducts includes dairy foods, where this compound is applied in the coloring of butter and ice-cream, maintaining a stable reddish color for up to four months [146,148,149].

Even though the use of the aforementioned compounds as food colorants could present an ecological solution to current production issues in addition to possessing an added advantage of potential health benefits, their use has been limited. Regulated colorants from natural sources include anthocyanins (E163), betanin (E162), and carotenoids (E160), including β-carotene (E160a), lycopene (E160d) (its obtention from tomato processing byproducts has been optimized), lutein (E161b), canthaxanthin (E161g), chlorophyll and chlorophyllin (E140 and E141), and curcumin (E100) [according to Regulation (EC) No 1129/2011] [163]. However, the list of anthocyanin colorants in the Codex Alimentarius includes only grape skin extract (E163), and in the FDA, “grape color extract” and “grape skin extract” (enocyanin) [146,148,149]. 

Regardless, to include a new pigment as a food colorant additive according to Regulation (EC) No 1333/2008 of the European Parliament and of the Council of 16 December 2008 [162], these new pigments need to be capable of restoring the original appearance of food whose color has been affected by processing, storage, packaging, and distribution, leading to impaired visual acceptability. Thus, colorants need to make food more visually appealing as well as give color to food that is otherwise colorless.

## 7. Texturizing Agents

Texturizing agents, such as emulsifiers, stabilizers, thickeners, and bulking agents, are used in the food industry to modify the overall texture and mouth feel of foodstuffs [171]. Thickeners, when added to the food mixture, increase the viscosity without modifying other food properties, while bulking agents increase the bulk of a food without affecting its nutritional value. Emulsifiers, on the other hand, allow water and oil to remain mixed together in an emulsion. These agents are used to add or modify the texture of food products by modifying the creaminess, thickness, viscosity, or by stabilizing foodstuffs structure [67]. These agents are used in frozen desserts, dairy products, cakes, puddings, gelatin mixes, dressings, jams, jellies, and sauces [172]. An example of the use of these food additives is their incorporation into hydrocolloids like fermented milks, dairy desserts, cream, and ice-cream to stabilize and thicken them. Another example of texture additives are phosphates and coagulation agents that are used in the curdling of milk in cheese production [173]. Most of the hydrocolloids used in the dairy industry result from the isolation of seaweeds and plant cells and are obtained and/or extracted from byproducts such as plant food wastes. The natural agar obtained from algae is the most researched texture agent used as a food additive in bakery products, confectionery, ice cream, peanut butter, and beverages [171]. Table 4 discloses some examples of byproducts used as source of texture additives.

Citrus fruits and their byproducts (such as peels and seed powders) have been studied as possible sources of texturizing agents due to their natural high pectin content and dietary fiber [142,176,180]. Currently, there are some examples of citrus byproducts being used in the industry. Oranges are being used as a texturizing agents in yogurts and/or ice creams [21,179,181], and lemon byproducts are being used as thickening, texturizing, gelling, and stabilizing agents [182]. Furthermore, citrus byproducts have the added advantage of being rich in bioactive compounds, which possess nutritional and functional benefits including reducing the risk of certain pathologies such as obesity, cardiovascular disease, and colon cancer, as well as preventing neurodegenerative diseases and osteoporosis [139,182,183,184,185]. Additionally, their high dietary fiber content is an added bonus, as it can be used as fat replacers and thus functions as a food additive to impart texture to the final product [186,187,188]. In fact, Crizel et al. [189] showed that incorporation of fiber from orange byproducts into yogurts allowed for the manufacture of low-fat yogurts, and Dervisoglu and Yazici [190] reported that while citrus fiber as a single stabilizer did not improve the viscosity, overrun, and sensory properties of ice cream, it improved the melting resistance of these foodstuffs. Similarly, the industrial processing of tomato leads to high amounts of unused matter (mostly peels and seeds), which are byproducts rich in lycopene and dietary fiber. These byproducts have been incorporated in tomato sauce as a food texturizer, with sensorial tasting panels deeming it as acceptable [149,191]. Another example is the ß-glucans resulting from cereals such as oat and barley, which have also been used as fat replacers in a variety of foodstuff ranging from baked goods and pasta to beverages and soups with promising results [192,193,194]. Additionally, the presence of β-glucans in foods has also been shown to lead to an increase in fiber intake, which in turn prevents constipation, reduces intestinal transit time, reduces the risk of colorectal cancer, and promotes the growth of beneficial intestinal bacteria [195].

Other potential sources of dietary fiber and pectin are cocoa (Theobroma cacao L.) pod husk (an abundant industrial waste with potential application in the food industry), and oat bran. Studies have shown that cocoa byproducts can be used as a texturizing agents after drying and grinding, while juice resulting from the pods can be used to prepare hydrocolloids [196]. On the other hand, oat bran extract has also been studied as a natural emulsifier, with results showing stability through a different range of pH values, heat treatments, and storage life up to 40 days [197].

On a different note, fat plays an important role in the structural integrity and mouth feeling of foodstuffs (ice-cream and yogurt in particular) due to its interaction with casein micelles [198]. Many different types of fat replacers have been explored in bovine and goat milk yogurts, including the addition of inulin, β-glucan, high milk protein powder, and whey protein concentrates [199,200,201,202,203,204]. Whey proteins, obtained as a byproduct of the dairy industry, have many functional properties including gelation, thickening, and water-holding capacity [205]. In the study by Wang et al. [206], whey protein isolate was used to produce a goat’s milk yogurt of acceptable quality. Milk fortification with whey protein improved the textural and microstructural characteristics and some sensory characteristics of yogurts. In addition, whey protein concentrates caused some interactions between globular proteins and caseins, which led to an improved texture of goat’s milk yogurt and higher water retention capacity [36,206,207,208]. Whey proteins are also present in high amounts in a byproduct of butter-making—buttermilk. This product is now considered valuable because of its high content in fragments of milk fat globule membrane in addition to phospholipids [209,210]. Studies indicated that the moisture content of cheese supplemented with buttermilk remained high due largely to phospholipids improving its texture [211,212].

## 8. Foaming Capacity

Foam is a colloidal dispersion in which a gaseous phase is dispersed in a liquid or solid phase. Food foams are dependent on the surface activity and film-forming properties of specific protein compounds [213,214]. Proteins have to be either very hydrophobic or hydrophilic to possess good foaming properties, and therefore their chemical or enzymatic modification can make them more active on the surface. As such, most foaming agents commonly used in the food industry are mainly natural modified food proteins such as soy, casein, egg white, whey, serum proteins isolated from lactoglobulins, and lysozyme [214,215,216]. Globulins are excellent foaming agents, but their foaming is significantly affected by interactions of the proteins with ovomucine, lysozyme, and, to a lesser extent, ovomucoid, ovotransferrin, and ovalbumin [217]. A novel source of possible foaming agents is the fishery industry, as fish processing leads to high amounts of byproducts rich in collagen and gelatin. This gelatin foaming capacity has been studied, with reports showing that gelatin from shark cartilage possessed foaming properties similar to those of porcine skin [218]. According to Muzaifa et al. [219], fish byproducts (dark muscle, cut offs, viscera, skin, scales, small bones, and fins) could potentially be used to obtain protein hydrolysates through an enzymatic hydrolysis using Alcalase^®^ 2.4 L and Flavourzyme^®^ 500 L, leading to compounds with foaming capacity. Protein hydrolysates obtained from poultry byproducts (head and leg) and rainbow trout (*Onchorhynchus mykiss*) viscera after an enzymatic hydrolysis using Alcalase^®^ 2.4 L also demonstrated foaming capacity [14]. On another work, Kotlar et al. [220] reported on the use of brewer’s spent grain (BSG) hydrolysates (attained using a *B. cereus* extracellular peptidase) to improve the foaming expansion in brewery products. Okara, a byproduct obtained from the soy milk production, was also analyzed for its functional properties, with the authors observing that the isolated proteins from this byproduct could potentially be used as a foaming agent [27]. When it comes to slaughter byproducts, there are several residues, including skin, bones, hooves, muscles, and blood. Blood represents up to 4% of the animal live weight. However, the direct use of blood in foods is not useful due to the dark color given to the food. In practice, blood is separated by centrifugation into cellular and plasma fractions. Plasma proteins have relevant and interesting properties for food processing [221], e.g., they contribute to cross-link proteins and gelling [222], proteins enrichment [223], as well as emulsifying and foaming agents [224]. 

## 9. Emulsifiers

Emulsifiers, molecules such as polysaccharides (e.g., gum arabic) or phospholipids (e.g., lecithins) with a surface activity capable of mixing and stabilizing two immiscible phases like water and oil, are largely used in food technology [225,226].

Emulsifier additives can be obtained from a variety of food products (e.g., milk protein isolates) [227] and byproducts (e.g., okara) [27]. Gbogouri, Linder, Fanni, and Parmentier [11] suggested that salmon (Salmo salar) head hydrolysates treated with the commercial enzyme Alcalase^®^ 2.4 L could potentially be a new source of compounds with great emulsifying capacity and stability. Using the same enzyme mix, Sathivel et al. [13] analyzed the potential of herring (Clupea harengus) byproducts hydrolysates. Although the emulsifying capacity was lower than that of egg albumin and soy protein, the hydrolysates still demonstrated some emulsifying capacity and stability, an effect that was also observed for the protein extracts before hydrolysis [12]. A potential emulsifier additive could be obtained from okara, a byproduct obtained from soymilk production. Even though okara protein isolates had poor solubility, they exhibited other functional properties (emulsification, foaming, and binding properties) that were comparable to those of a commercial soy isolate, further demonstrating the potential use of these isolates as a food ingredient [27]. The Horchata production, a vegetable milk obtained from tiger nuts (Cyperus esculentus), also originates a solid waste byproduct rich in dietary fiber that could potentially be used as a new ingredient for its emulsifying capacity and high emulsifying stability [28]. Emulsifiers can also be found in meat industry byproducts. For example, bovine blood derivate products (plasma and globulin) may be used as a potential new emulsifier agents additive in meat products and others [28,228,229]. As such, compounds obtained directly or indirectly from byproducts could potentially be used as new emulsifying agents in the food industry.

## 10. Conclusions

Given the consumer’s demand for “clean label” products and the environmental constraints that reinforce the need to change the traditional industrial raw matters with renewable sources, agro-food byproducts have appeared as one of the most relevant potential solutions. In fact, some of the additives used nowadays (like anthocyanins and carotenes) can be found in these materials, which makes their extracts interesting from a consumer’s perspective (some would prefer a tomato extract instead of traditional lycopene), particularly when considering the possibilities opened up by green, safe, new extraction methodologies like high pressure extraction, ohmic extraction, pulsed electric field, or supercritical extraction. However, their direct inclusion into commercial products may depend on the limitations posed by the legislation itself because, even if the additive itself is already approved for use, should its production process or raw material differ significantly from the one currently used, its future as an additive will be dependent on a new safety evaluation.

Overall, it is possible to see the potential of byproducts derived food additives and potential new additives for application in the food industry. They are an integrated solution with low cost and reduced environmental impact capable of providing alternatives for an industry that relies heavily upon the chemical synthesis compounds. Thus, the use of byproducts as a source of food additives stands out as an economically and environmentally conscious choice and will promote the new era of circular economy. 

## Figures and Tables

**Table 1:** Potential applications of agro-food byproducts compounds.

Byproduct	Origin	Compounds	Bioactivity	Types of Extract	Dose of Byproduct Extract	Reference
**Buffalo horn**	Animal	Peptides	Antioxidant	Aqueous		[8]
**Bovine Achilles tendon collagen**	Animal	Peptides	Antihypertensive	Aqueous		[9]
**Bovine haemoglobin hydrolysate**	Animal	Peptides	Antibacterial Antihypertensive	Ethanolic	187.1 and 35.2 μM 42.55 to 1095 μM	[10]
**Salmon fish**	Animal	Hydrolysates	Emulsifying capacity Emulsion stability Fat adsorption capacity	Aqueous	0.02–5% mg/mL	[11]
**Herring fish**	Animal	Protein	Emulsifying capacity Emulsion stability Fat absorption capacity	Aqueous	5–50 mg/mL	[12]
		Hydrolysates	Emulsifying capacity Emulsion stability Fat adsorption capacity Antioxidant activity	Aqueous and ethanolic	0.01–50 mg/mL	[13]
**Poultry**	Animal	Hydrolysates	Water holding capacity Oil absorption capacity Emulsifying capacity Foaming capacity	Aqueous	7.5–100 mg/mL	[14]
**Rainbow trout viscera**	Animal	Hydrolysates	Water holding capacity Oil absorption capacity Emulsifying capacity Emulsion stability Foaming capacity	Aqueous	7.5–100 mg/ml	[14]
**Grape peels**	Fruit	Phenolic compounds Anthocyanins	Antioxidant	Ethanolic	1.652 mg/mL	[15]
**Pomegranate seed**	Fruit	Phenolic compounds	Antioxidant	Ethanolic	25 mg/L	[16]
**Pineapple peels powder**	Fruit	Dietary fibres	Texturizing agent	Aqueous	1%	[17]
**Banana peels**	Fruit	Flavonoids. Tannins B-carotene	Antioxidant	Aqueous	5 mg of banana peel extracts/1 mL	[18]
**Grape seed**	Fruit	Phenolic compounds Flavonoids	Antioxidant Antimicrobial	Ethanolic	100 mL/25 g	[19]
**Wine pomace, skin and seed extracts**	Fruit	Total Phenolic Flavonoid Compounds	Source of phenols	Ethanolic	0.1, 0.2 and 0.3 wt/vol	[20]
**Orange byproducts**	Fruit	Dietary fibres	Texturizing agent	Aqueous	0.2 to 1 g/mL	[21]
**Wine pomace extract and flour**	Fruit	Dietary fibres Phenolic compounds	Antioxidant Colorant	Ethanolic	1% to 3% 1% to 2%	[22]
**Wine pomace flour**	Fruit	Polyphenols Prebiotics Phenolic compounds	Source of phenols, Probiotic protection	Aqueous	10, 20 and 50 g/L	[23]
**Grape seed**	Fruit	Phenolic acids Flavonoids Aromatic compound	Antioxidant Antimicrobial	Ethanolic	10 mL/25 g	[19]
**Banana inflorescence bracts**	Fruit	Anthocyanin cyanidin-3-rutinoside	Antioxidant Colorant	Ethanolic	14–32 mg/100 g	[24]
**Pear stones**	Fruit	Dietary fibres	Texturizing agent	Aqueous	3–5%	[25]
**Male flower (*Musa paradisiaca*)**	Vegetable	Epigallocatechin and derivatives	Antioxidant Antimicrobial	Ethanolic Aqueous	12.93–2.34%	[26]
**Soy milk**	Vegetable	Protein	Water and fat binding capacity Foaming capacity Emulsifying capacity Emulsion stability	Aqueous		[27]
**Tiger nut milk**	Vegetable	Dietary fibres	Water and oil holding capacity Water and oil absorption capacity Emulsifying capacity Emulsion stability	Aqueous	1.5% mg/mL	[28]
**Tomato byproducts**	Vegetable	Dietary fibres, Proteins, Carotenoids, Tocopherols, Polyphenols Lycopene	Anti-inflammatory, Antiallergenic, Antimicrobial, Vasodilatory, Antithrombotic, Cardioprotective Antioxidant Colorant	Ethanolic	55.70 to 28.64 mg/100 g	[29,30]
**Overly ripe berries**	Vegetable	Phenolic compounds Dietary fibres	Antioxidant Anti-inflammatory Antimicrobial Antidiabetic Neuroprotective	Ethanolic		[31]
**Pomegranate peel**	Vegetable	Phenolic compounds Proanthocyanidins Tannins Flavonoids Oligomeric ellagitannins	Antimicrobial Antioxidant	Aqueous-methanolic	5-g portions in 80% methanol Antimicrobial activity tested at 0.1% (*v*/*v*)	[32,33]
**Tomato processing byproduct**	Vegetable	Lycopene Phenolic compounds	Antioxidant	Ethanolic	400 and 800 mg/kg	[34]
**Rice bran**	Cereal	γ-oryzanol Anthocyanins Phenolic compounds	Colorant	Ethanolic	0.2–0.6%	[35]
**Whey protein**	Dairy products	Caseinates	Texturizing agent Source of protein	Aqueous	2%	[36]

**Table 2:** Types of food additives and their functions as described by European Food Safety Agency (EFSA) in Regulation (EC) No 1333/2008.

	Function
Sweeteners	Increase the sweetness (can be added or table-top)
Colors	Add or restore color
Preservatives	Prolong shelf-life by inhibiting microbial deterioration or the growth of pathogens
Antioxidants	Prolong shelf-life by inhibiting oxidative deterioration (e.g., color changes or rancidity)
Carriers	Physically modify a compound to ease its application/handling, without compromising the activity of the added compounds and having no technological effect by themselves
Acids	Increase the acidity and/or impart a sour taste
Acidity regulators	Alter/control the pH of a foodstuff
Anti-caking agents	Reduce particle agglomeration
Anti-foaming agents	Prevent/reduce foam formation
Bulking agents	Increase the volume of a foodstuff without significantly increasing its energetic value
Emulsifiers	Ease the formation/maintenance of an homogenous mixture of two immiscible phases
Emulsifying salts	Convert cheese proteins into a dispersed form contributing to an homogenous distribution of other components (e.g., fat)
Firming agents	Either keep fruit and vegetables firm/crisp or produce/strengthen gels
Flavor enhancers	Enhance taste/odor
Foaming agents	Ease the dispersion of a gaseous phase in a liquid/solid
Gelling agents	Form a gel and improve texture
Glazing agents (including lubricants)	Give a shiny appearance or provide protective coating
Humectants	Prevent drying or promote the dissolution of powders in an aqueous media
Modified starches	Chemically treated edible starches
Packaging gases	Gases (not air) introduced into containers before placing the foodstuff in them
Propellants	Gases (not air) that expel a foodstuff from a container
Raising agents	Release gas therefore increasing the volume or a dough or batter
Sequestrants	Complex metallic ions
Stabilizers	Maintain the physico-chemical state of a foodstuff
Thickeners	Increase the viscosity
Flour treatment agents	Improve the baking quality of flours/doughs (non-emulsifiers)

**Table 3:** Food byproducts sources of potential colorant food additives. Adapted from Iriondo-DeHond, Miguel, and del Castillo [142].

Food Industry	Byproducts	Function	Color	Chromophore	Reference
**Winery**	Wine pomace extract and flour	Antioxidant and colorant agent	Red to purple to blue	Anthocyanins	[22]
**Cereal**	Rice bran	Colorant agent	Yellow to light brown	Carotenoids	[35]
**Vegetable**	Lycopene from tomato byproducts	Colorant agent, antioxidant, and antimicrobial	Red	Carotenoids	[148]
	Carotenoids from tomato peels	Antioxidant and colorant agent	Red	Carotenoids	[149]
	Phenolic compounds from eggplant	Colorant agent, antioxidant, and antimicrobial	Purple-blue	Anthocyanins	[130]
	Phenolic compounds from potato peels	Colorant agent, antioxidant, and antimicrobial	White	Anthoxanthins	[150]
	Leafy green vegetables	Colorant agent and antioxidant	Green	Chlorophyll	[151]
**Fruit**	Anthocyanins from berries’ peels Phenolic compounds from tropical and citrus fruits peels, seeds, and unused flesh	Colorant agent, antioxidant, and antimicrobial	Purple-blue Yellow-orange	Anthocyanins Carotenoids	[33,131,133,152,153,154]

**Table 4:** Food byproducts sources of potential texturizing agents’ additives. Adapted from Iriondo-DeHond, Miguel and del Castillo [142].

Food Industry	Byproducts	Function	Reference
**Fruit**	Olive pomace	Texturizing agent	[142,174,175]
	Passion fruit peels		[142,174,175]
	Grape pomace		[142,174,175]
	Pineapple peel powder		[17]
	Pineapple fruit peels		[176]
	Orange byproducts		[21,177]
	Citrus peels		[142]
	Apple pomace		[142]
	Pear stones		[25]
**Dairy**	Whey protein		[36,178]
	Whey protein and Buttermilk		[179]
**Vegetable**	Onion hulls		[142]
	Spinach		[25]
